# Influence of Nodal Spheres on the Mechanical Behaviour of Auxetic Materials Manufactured with PA12

**DOI:** 10.3390/ma18245688

**Published:** 2025-12-18

**Authors:** Ismael Lamas, Iria Feijoo, Silvia Gómez, Alejandro Pereira, José A. Pérez, M. Consuelo Pérez

**Affiliations:** 1 Cintecx, University of Vigo, Encomat, 36310 Vigo, Spain; 2 Materials Engineering Department, Industrial Engineering School, University of Vigo, 36310 Vigo, Spain; ifeijoo@uvigo.es (I.F.); sgomez@uvigo.es (S.G.); 3 Design Engineering Department, Industrial Engineering School, University of Vigo, 36310 Vigo, Spain; apereira@uvigo.es (A.P.); japerez@uvigo.es (J.A.P.)

**Keywords:** cellular material, auxetic metamaterial, selective laser sintering, polyamide 12, nodal spheres

## Abstract

Auxetic metamaterials, characterised by a negative Poisson’s ratio, offer excellent energy absorption but often present limited compressive strength due to their strut-based architectures. Selective laser sintering (SLS) enables the precise fabrication of these structures, yet enhancing their mechanical performance remains challenging. This research investigates the influence of nodal spheres on re-entrant dodecahedral unit cells produced in PA12, varying node-to-strut diameter ratios (1:1, 2:1, and 3:1). Compression tests reveal significant increases in stiffness and compressive strength, reaching up to 88.70% for the 3:1 ratio. When normalised by relative density, the 2:1 configuration proves most effective, achieving a 35.33% increase in specific strength and a 19.58% improvement in specific energy absorption. The deformation behaviour indicates a mixed bending–stretching mechanism, with geometry exerting a stronger influence than the base material. Although larger nodal spheres enhance absolute strength, they also increase mass and relative density, which may limit their suitability for weight-sensitive applications. Overall, these findings highlight nodal reinforcement as a promising strategy to enhance the mechanical efficiency of auxetic metamaterials while maintaining their auxetic response. These improvements support applications in aerospace, automotive engineering, personal protection systems, lightweight structural panels, and energy-absorbing components.

## 1. Introduction

In the field of materials engineering, auxetic materials are among the main mechanical metamaterials [1], characterised by their unique behaviour of exhibiting a negative Poisson ratio. Auxeticity, which results from precisely engineered internal microstructures, grants auxetic materials properties such as enhanced indentation resistance [2,3,4], increased shear stiffness [5,6,7,8], and improved energy absorption capacity [1,9,10,11]. These features have made auxetic materials a focal point of research for applications where impact resistance, energy dissipation, and structural resilience are critical.

In the automotive industry, for instance, auxetic structures can be incorporated into components designed to absorb energy, such as bumpers or crumple zones. This improves crash resistance and occupant safety [12,13,14]. In the aerospace sector, lightweight auxetic materials with high energy absorption potential could enhance the impact resistance of various components without significantly increasing weight. Similarly, auxetic metamaterials are attracting increasing interest for use in biomedical applications, where they can be used to design implants or prostheses that adapt more effectively to physiological movements and forces while protecting surrounding tissues from sudden impacts [15,16,17,18]. Beyond these, auxetic lattices are increasingly explored for vibration-damping components, protective housings in renewable-energy systems, and lightweight structural panels.

Interest in auxetic materials has grown significantly since the pioneering study of R.S. Lakes in 1987 [7], which established auxeticity as a valuable mechanical property and stimulated the development of new metamaterial designs for diverse industrial applications. A key advantage of auxetic materials is their high energy-absorption capacity: under impact or compression, their deformation mechanisms enable efficient stress redistribution and effective energy dissipation [19,20,21,22,23]. Cellular auxetic metamaterials achieve their negative Poisson ratio through re-entrant, chiral and rotational lattice geometries [24], which densify under load and concentrate material in the bearing region, enhancing impact mitigation. However, these same deformation mechanisms can compromise strength and stiffness, making it necessary to balance energy absorption with mechanical performance to broaden their practical use [25,26,27].

Recent research has consistently demonstrated that incorporating nodal reinforcement into lattice architectures significantly enhances their mechanical performance and energy-absorption capacity. For instance, in metallic lattices fabricated by additive manufacturing, node-reinforced diamond lattice structures exhibited higher yield strength, collapse strength and energy absorption compared to non-reinforced lattices under quasi-static compression, while also showing more uniform stress distribution and reduced porosity [28,29]. Similar improvements have been reported in polymeric and graded lattices, where node-enhanced BCC (NBCC) structures used in automotive crash-energy-absorbing cores achieved up to a 30% increase in specific energy absorption and substantial reductions in peak impact forces under drop-hammer and SHPB loading [30]. Homogenisation and numerical studies corroborate these trends, showing load-bearing increases of ~25% in NBCC lattices compared to their non-reinforced counterparts [31]. Moreover, a recent review on additively manufactured lattice structures highlights that nodal geometry (shape and size of junctions) is among the most influential design features affecting strength, stiffness, energy absorption, and fatigue resistance across different materials and topologies [32,33]. These observations are aligned with prior works indicating that nodal reinforcement can improve stiffness, delay failure and enhance dynamic energy absorption by reducing stress concentrations at strut junctions, and tensile studies on TPMS-based nodal modifications further demonstrate gains in strength and damage tolerance across multiple matrix materials [34]. Comprehensive reviews also identify nodal geometry as one of the most influential design parameters governing fatigue resistance, robustness and overall mechanical efficiency in architected materials [35]. Collectively, these findings underline the critical role of nodal reinforcement as a design strategy, particularly relevant for auxetic metamaterials, where the influence of nodal geometry on negative-Poisson deformation mechanisms and energy absorption remains comparatively underexplored.

However, despite extensive research on nodal reinforcement in conventional lattice structures, very limited attention has been given to how nodal geometry interacts with the deformation mechanisms that govern negative-Poisson-ratio behaviour. In particular, no experimental studies have systematically evaluated how the relative size of nodal spheres influences the mechanical performance and energy absorption of auxetic lattices. This gap is especially relevant because auxetic metamaterials concentrate deformation differently from non-auxetic lattices, and the role of nodal reinforcement in these distinctive kinematic patterns remains largely unexplored.

Polyamide 12 (PA12) is an aliphatic polyamide widely used in Selective Laser Sintering (SLS) due to its low melting point, reduced hygroscopicity, and balanced mechanical and chemical properties. Its durability, flexibility at low temperatures, chemical resistance, and low friction make it suitable for producing complex metamaterial geometries. SLS, which uses unsintered powder as a self-supporting medium, enables the fabrication of intricate lattices and auxetic structures without support material, making it ideal for architected cellular systems.

Given the importance of optimising topological parameters to influence deformation mechanisms and improve the performance of auxetic metamaterials, this paper will analyse the relationship between nodal sphere diameter and lattice strut diameter, as this is a critical design parameter that directly affects deformation behaviour, energy absorption, and overall mechanical performance. Optimising this ratio could lead to auxetic structures with greater strength and energy dissipation without compromising their inherent auxetic properties. However, adding nodal spheres can considerably increase the mass or relative density of the structure, which directly impacts material efficiency. Therefore, this paper will conduct an experimental evaluation of three auxetic structures manufactured from polymeric material with different sphere-to-strut ratios to identify the structure with the best mechanical properties and the least material use, thus maximising energy absorption efficiency without adding unnecessary weight.

The aim of this research is to provide empirical data to contribute to the efficient design of these innovative structures and improve our understanding of the impact of nodal reinforcement on their strength and energy absorption efficiency.

## 2. Materials and Methods

### 2.1. Design and Structures

The specimens used in this study were designed with a cubic configuration, incorporating an internal cellular structure and two external plates to form a sandwich panel geometry. The cubic shape and sandwich panel configuration were chosen to optimise the use of the Sinterit Lisa printer’s (Sinterit Sp. z o.o., Kraków, Poland) maximum build volume of 110 mm × 160 mm × 130 mm (X, Y, Z). Consideration was also given to the standard to be followed for the compression tests: UNE-EN ISO 844:2021 [36], which specifies a minimum specimen area of 2500 mm^2^. To maximise the number of printed specimens and minimise the number of print runs, the dimension available along the X-axis and a minimum spacing of 3 mm were considered. This resulted in specimens with a side length of 51 mm. The external plates of the sandwich panel were modelled to face outwards, thereby ensuring that the cellular structure maintains uniform dimensions across all axes. This orientation avoids the reduction in cellular material height that would occur if the plates were modelled inwards. The thickness of the external plates was set equal to the strut diameter (1.275 mm). This design choice ensures a uniform mass distribution throughout the specimen and avoids abrupt transitions in thickness, which are known to concentrate stresses and negatively affect print quality in selective laser sintering (SLS). Maintaining consistent wall and feature thicknesses is a widely recommended SLS design guideline, as it promotes homogeneous energy absorption during laser exposure and reduces the likelihood of differential cooling, warping, or dimensional inaccuracy. By matching the plate thickness to the strut diameter, the sandwich panel achieves balanced stiffness across its boundaries while preserving geometric coherence with the internal cellular structure.

**Figure 1:**
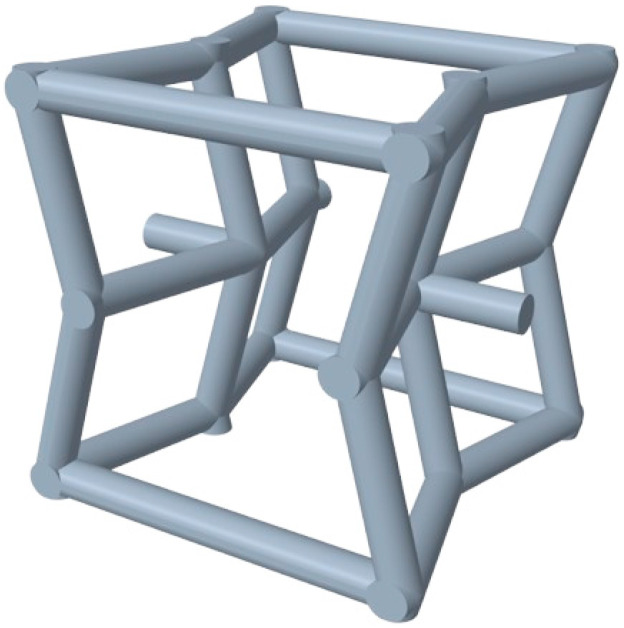
Re-entrant dodecahedral cell, available on Creo Parametric Software.

The rise in auxetic materials in recent decades has introduced new challenges to the modelling of cellular structures. Most software packages either require the prior design of the unit cell or rely on implicit modelling (i.e., mathematical equations), thereby increasing the complexity of metamaterial design. However, Creo Parametric software version 10.0.0.0 (PTC Inc., Boston, MA, USA), released in 2023, incorporates predefined auxetic cells, including the re-entrant dodecahedral cell. This allows them to be applied directly to complex structures. This cell is available in two variants: the full re-entrant dodecahedral version (with re-entrant angles in two directions) and a simplified version (with re-entrant features in only one direction). This study employs the full version, as shown in Figure 1. This version offers greater flexibility for modifying geometric parameters and facilitates the design of various metamaterials through the combination of angles. The software was used under an educational licence for students.

This type of re-entrant dodecahedral cell is derived from re-entrant hexagonal structures and is optimally suited to energy absorption applications. It incorporates additional struts while maintaining a bending-dominated deformation mechanism. This is confirmed by applying Maxwell’s stability criterion [37], which for three-dimensional (3D) lattices follows Equation (1). If *M* is less than zero, the topology’s deformation mechanism is bending-dominated. This deformation mode is ideal for damping applications [38,39,40].

(1) M=b−3j+6=30−3×18+6<0,

where

*M* is the coefficient;

*b* is the number of struts;

*j* is the number of nodes.

A unit cell size of 12.75 mm × 12.75 mm × 12.75 mm was adopted to maximise the dimensions of the unit cell while ensuring enough cells were present to induce auxetic behaviour. Previous studies have shown that similar structures begin to exhibit a negative Poisson ratio after three repetitions of the unit cell [41].

The strut diameter is 1.275 mm, corresponding to 10% of the cell dimension. This yields a low relative density, enabling a more effective evaluation of the geometric influence of the cell structure. In materials with a high relative density, mechanical properties tend to converge regardless of the topology employed, as these properties become increasingly dependent on the base material used to fabricate the metamaterials [42,43]. This diameter also aligns with the manufacturer’s recommendation of using wall thicknesses greater than 0.5 mm to ensure material durability during post-processing.

**Figure 2:**
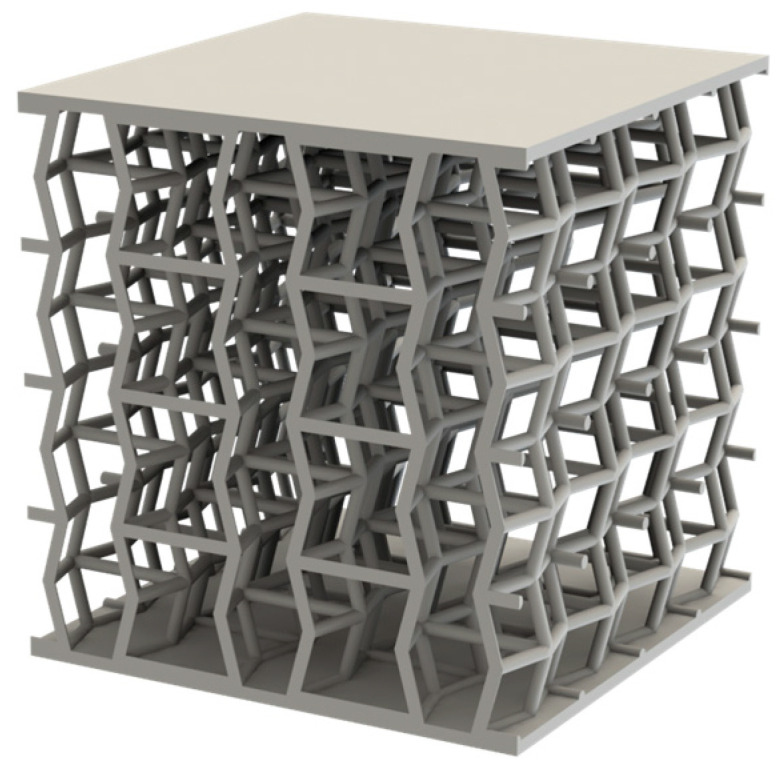
Rendering of the auxetic specimen with a ratio of 1, produced using the SolidWorks tool PhotoView 360.

Once these parameters had been set, the geometry shown in Figure 2 was created using SolidWorks (version 2022–2023) with the PhotoView 360 module, for which an academic licence was provided by Dassault Systèmes (Vélizy-Villacoublay, France) to students. These specimens are considered the reference configuration, as they do not include nodal spheres (node-to-strut diameter ratio = 1).

**Figure 3:**
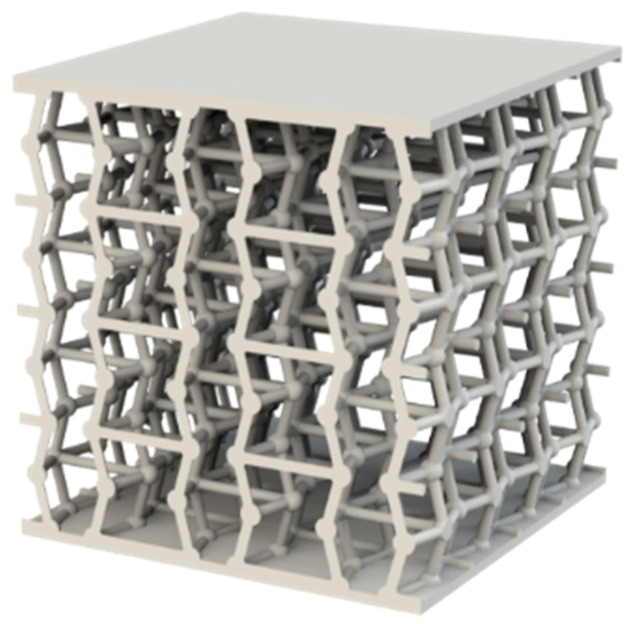
Rendering of the auxetic specimen with a 2:1 ratio, produced using the SolidWorks tool PhotoView 360.

**Figure 4:**
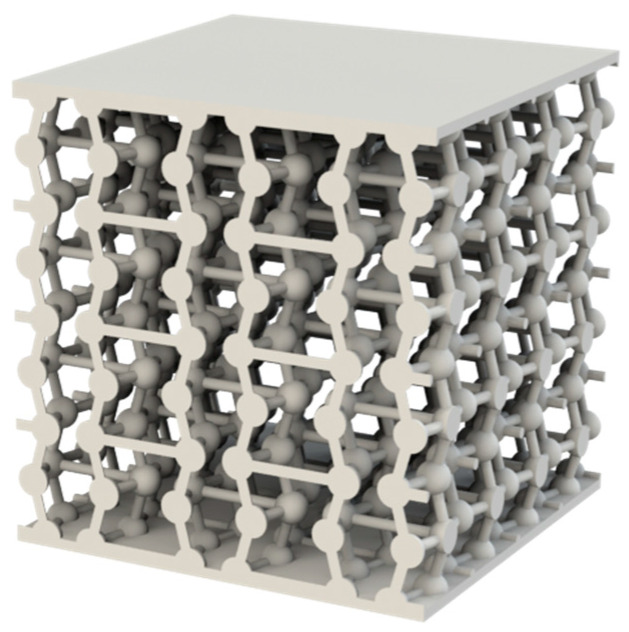
Rendering of the 3:1 ratio auxetic specimen made with the SolidWorks tool PhotoView 360.

Regarding the nodal spheres, the specimens with a 2:1 ratio (Figure 3) have a sphere diameter of 2.55 mm, whereas the specimens with a 3:1 ratio (Figure 4) have a diameter of 3.825 mm.

These design parameters ensure that the specimens meet the mechanical requirements necessary for evaluation in compression tests, enabling their energy absorption and structural strength properties to be characterised in detail. In summary, the aim of the specimen design was to maximise manufacturing efficiency by optimising dimensions and selecting a suitable auxetic cell, while ensuring compliance with compression test standards and minimising defects associated with additive printing.

To evaluate whether introducing nodal spheres into metamaterial joints can improve their mechanical properties without compromising their auxetic properties, a finite element method (FEM) analysis was carried out. This analysis provides a preliminary theoretical estimation of the mechanical behaviour of the various structural configurations prior to fabrication, thereby facilitating decision-making regarding the printing of the specimens. The analysis is performed using the ANSYS (2022 R2) software, provided by Ansys, Inc. (Canonsburg, PA, USA), in its structural static analysis mode. The material was defined using the experimentally obtained properties from previous tests, with a density of 0.8949 g/cm^3^, a Young’s modulus of 481.46 MPa, and a Poisson ratio of 0.20. A linear elastic isotropic model was used, as the goal was to analyse stress concentration without reaching the plastic region.

In all cases, the same boundary conditions were applied: the bottom face of the structure was fixed, and an arbitrary downward vertical displacement of 10 mm was applied on the top face.

The structures were discretised using 10-node quadratic tetrahedral elements (SOLID187, ANSYS Workbench default for mechanical analyses), with an element size of 0.5 mm, applying the same meshing strategy across all specimens to ensure comparability.

The final mesh size depended on the node-to-strut diameter ratio. The 1:1 configuration consisted of 722,063 nodes and 362,445 tetrahedral elements, the 2:1 configuration of 758,299 nodes and 386,483 elements, and the 3:1 configuration of 887,736 nodes and 469,383 elements.

Although the von Mises equivalent stress was used as the evaluation metric, its role in this study was mainly qualitative. The onset of peak stress in an individual beam does not imply the failure of the metamaterial as a whole, since the structure exhibits a distributed load-bearing behavior and its global response is governed by the collective deformation of the unit cell rather than by the rupture of a single element. In this context, local stress concentrations are expected and were interpreted as indicators of how the load is redistributed through the re-entrant geometry, rather than as absolute failure thresholds. For this reason, the stress field was analysed to understand the pathways of load transfer within the structure, instead of extracting definitive failure values.

Throughout the study, different structural configurations are compared. These include a base structure without nodal spheres, as well as structures with nodal spheres equivalent to two or three times the diameter of the struts. The Von Mises equivalent stress distribution is analysed in all of the structures as a criterion for evaluating stress concentration at critical points.

**Figure 5:**
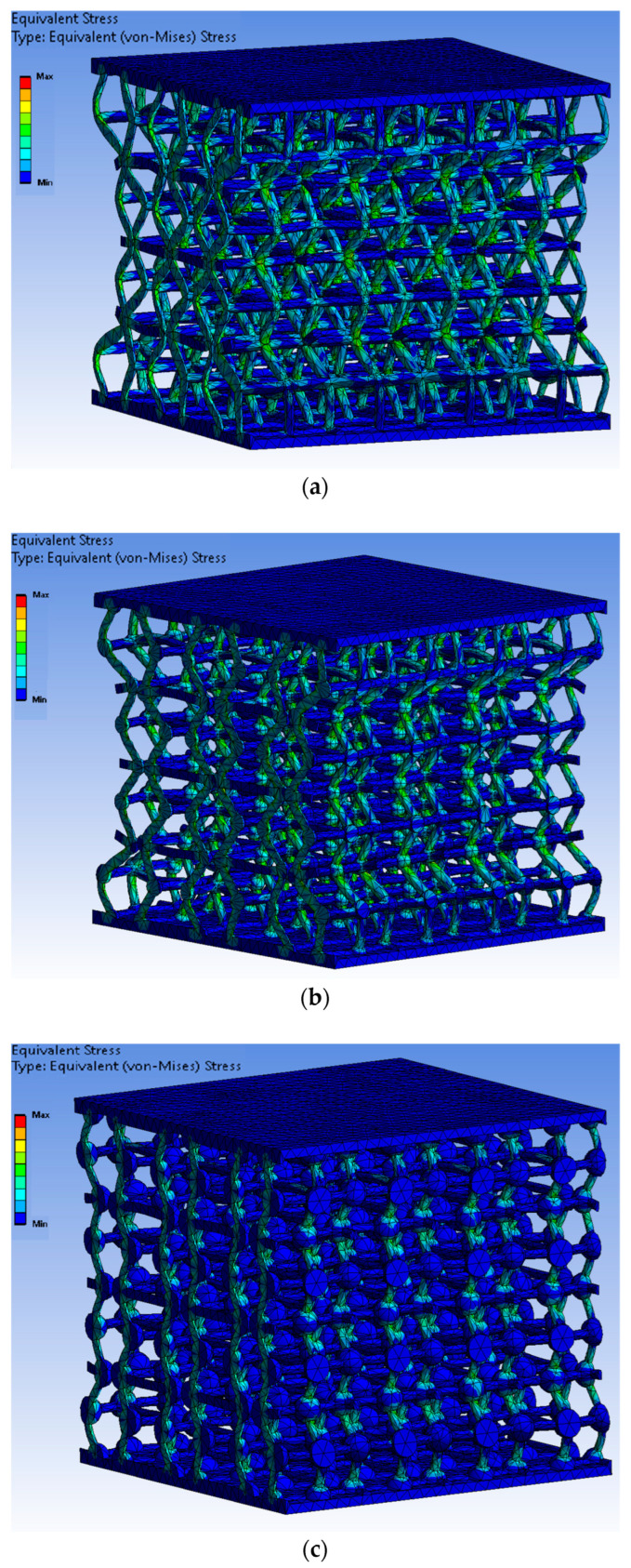
Stress map of specimens obtained, created using ANSYS Workbench: (**a**) without nodal spheres (ratio 1:1); (**b**) with nodal spheres with diameter 2 times the strut diameter (ratio 2:1); (**c**) with nodal spheres with diameter 3 times the strut diameter (ratio 3:1).

Figure 5 shows the results obtained from the FEM simulation for all types of specimens. Thus, the inclusion of nodal spheres reduces stress in the strut connections. Furthermore, increasing the diameter of the nodal sphere progressively decreases the maximum stress in these areas. This decrease in stress concentration suggests a more favourable stress distribution, which could result in an increase in the metamaterial’s structural strength and durability.

Overall, the FEM analyses suggest that introducing nodal spheres is a promising mechanical strategy for reducing stress concentrations in the strut joints. Furthermore, these modifications do not affect the auxetic behaviour of the structures, and they maintain their capacity for transverse negative deformation under uniaxial loading.

### 2.2. Specimens Manufacture

The manufacture of the metamaterials was performed by selective laser sintering (SLS). The selected material was polyamide 12 (PA12), commonly known as Nylon 12. This polymer presents a good chemical resistance, high flexibility at low temperatures, and good dimensional stability, making it suitable for a wide range of applications. PA12 Smooth v2 (Sinterit, Poland) was chosen from Sinterit’s PA12 options, as it offers a better surface finish and a higher level of detail in prints.

The Sinterit Studio v1.10.0 software was used for lamination and print setup. The 3MF format was selected for transferring the CAD file to the CAM system due to its lower computational cost and ability to store detailed print information. The layer height was set to 0.125 mm to achieve an intermediate print quality without extending production times. This layer height corresponds to around 10% of the strut diameter (1.275 mm), ensuring each strut is reproduced with sufficient vertical resolution and without excessive staircase effects. This maintains the geometric fidelity required for auxetic lattice structures. Meanwhile, the print bed temperature was kept at 175.5 °C, slightly below the material’s melting point, to encourage adhesion of the unsintered powder. This powder acts as a support for the structure during printing.

**Figure 6:**
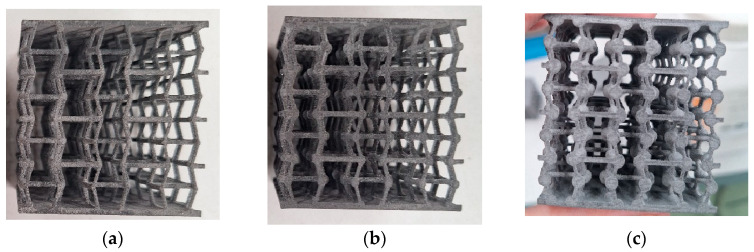
Specimens obtained by SLS: (**a**) ratio 1:1; (**b**) ratio 2:1; (**c**) ratio 3:1.

Three different metamaterials (ratio nodal sphere diameter/strut diameter: 1:1, 2:1, and 3:1) were printed, obtaining four specimens of each type. Figure 6 shows one specimen of each metamaterial.

### 2.3. Morphological Analysis

To analyse the surface condition of the specimens and the possible existence of impression defects, a morphological analysis was performed using a JEOL JSM-6510 scanning electron microscope (JEOL Ltd., Tokyo, Japan) equipped with an energy-dispersive spectrometer (EDS) and INCA Energy software (Version 4.13, Oxford Instruments, Oxford, UK). Thus, the study was carried out before and after mechanical testing to assess the integrity of joints between struts and spheres, and to characterise the fracture surfaces of 3D printed specimens. Secondary electron images were taken with a voltage of 20 kV, and the specimens were coated with a thin layer of Au using ion sputtering to improve the conductivity and minimise the charging effects.

For each node-to-strut diameter ratio (1:1, 2:1, and 3:1), a random sample of specimens was performed, focusing the observations on the node–strut joint, as it represents a critical area associated with a change in cross-section. These geometric transitions can generate phenomena such as thermal gradients, insufficient sintering, or uneven cooling, which could promote the appearance of defects. The selection of this area allowed the evaluation of possible defects by the section change such as porosity, lack of bonding, or dimensional distortions that could compromise the quality of the print and, consequently, the integrity of the joint. Objective criteria were used to define defects, considering as such any discontinuity associated with incomplete particle coalescence, voids larger than the local particle diameter, micro-cracks not attributable to the main fracture path, and irregular sintering zones deviating from the expected surface morphology.

### 2.4. Compression Test

The characterisation of the mechanical properties of the metamaterials was conducted through the implementation of compression tests, in accordance with the UNE-EN ISO 844:2021 standard for rigid cellular plastic materials. The determination of compression characteristics is achieved through the measurement of compressive strength. This document establishes that the first maximum stress corresponds to the compressive strength.

The experimental tests were conducted using a Shimadzu AGS universal testing machine (Shimadzu Corporation, Kyoto, Japan), which possesses a load capacity of 250 kN and has been adapted for the execution of compression tests. During the tests, the moving frame applied a downward displacement, and the specimens were placed on the lower fixed plate to ensure alignment and load stability. The control of the machine and the calculation of the mechanical properties of the structures was achieved by means of the Trapezium X software.

## 3. Results and Discussion

### 3.1. Morphological Analysis

**Figure 7:**
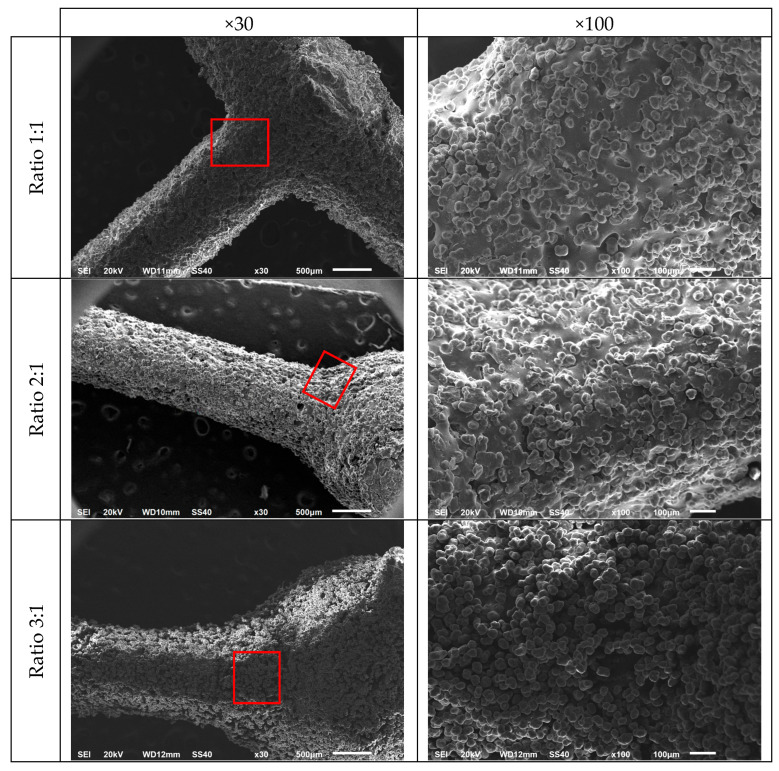
Scanning electron microscopy images of the strut joints of three different specimens (Ratio 1:1, Ratio 2:1 and Ratio 3:1) at 30× and 100× magnification. Red square is the magnification area.

Figure 7 presents SEM images of the SLS-fabricated specimen surfaces, corresponding to Ratio 1:1, Ratio 2:1 and Ratio 3:1 specimens, at 30× and 100× magnification, respectively.

The micrographs obtained for all probes show no significant manufacturing defects. Furthermore, no notable differences were identified among the various node-to-strut diameter ratios, suggesting consistent fabrication across all configurations. The strut connections exhibited uniform and well-defined interfaces, both in the presence and absence of nodal spheres.

**Figure 8:**
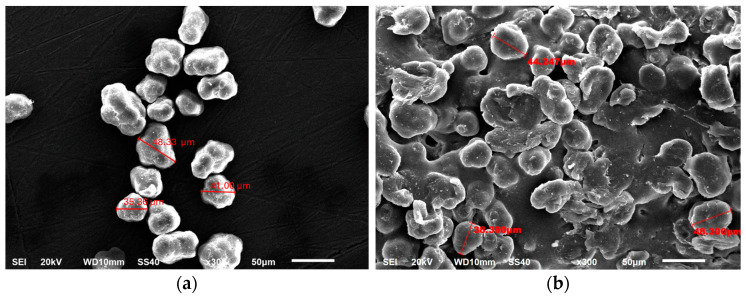
Morphology of the powders in the virgin state (**a**) and after fabrication (**b**).

Figure 8 compares the morphology of the powders in their virgin state (Figure 8a) and after fabrication (Figure 8b). As can be seen in Figure 8a, the micrograph shows particles that are relatively spherical and similar in size to those shown in Figure 8b.

The virgin powder particles are spherical, with an average grain diameter of 40 µm. Figure 8b shows spherical powder particles with a similar diameter to that measured for the virgin powder. This suggests that the surface of the specimens exhibits characteristics associated with melted PA12 particles, as well as submerged features in the melted PA12 matrix. These features include unmelted PA12 particle cores, which are particularly prevalent in the larger nodal spheres. Consequently, the surrounding powder (acting as a bed) adheres more readily to the spheres’ surface. However, the surface roughness generated by unmelted particles does not affect the properties of the parts and is typical of this manufacturing process [44,45]. This phenomenon could be explained by the laser remaining in contact with the spheres’ surface for a longer duration, thereby sintering a greater volume of material.

### 3.2. Compression Tests

The compression properties of the designed auxetic metamaterials were evaluated and the influence of the nodal spheres analysed by testing four specimens of each metamaterial.

**Figure 9:**
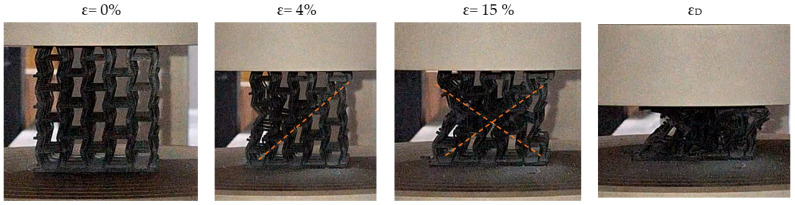
Sequential representation of deformation behaviour of specimen with 1:1 ratio. The orange dashed lines indicate the dominant deformation bands formed during compression tests.

**Figure 10:**
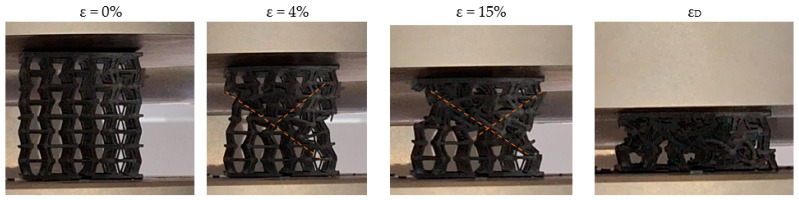
Sequential representation of deformation behaviour of specimen with ratio 2:1. The orange dashed lines indicate the dominant deformation bands formed during compression tests.

Firstly, the macroscopic compression behaviour of the specimens was analysed. Figure 9 and Figure 10 show sequential representations of the deformation behaviour of two specimens, one with and one without nodal spheres, corresponding to a 2:1 ratio, respectively. The sequence of micrographs ranges from an initial strain value of ε = 0% to densification deformation. As can be seen, the deformation mechanism remains similar in both specimens.

The deformation sequence can be described as follows: When the load is first applied, the auxeticity of the material becomes evident, resulting in deformation predominantly governed by bending. This behaviour persists until the maximum stress is reached simultaneously in several struts, particularly those forming re-entrant angles. After this point, the fracture mechanism becomes more similar to the stretch-dominated type.

In the metamaterial devoid of nodal spheres, the initial fracture propagates diagonally, and the subsequent primary fracture occurs in an analogous way, intersecting the previously formed diagonal and thereby generating an X-shaped fracture geometry. In specimens with nodal spheres, the initial fracture displays a propagation pattern that is analogous, albeit with minor variations that become more pronounced as the fracture progresses, particularly in its nascent stages. This fracture demonstrates a geometry more akin to a Y-type fracture than an X-type fracture. The disparities in the geometry of the fracture mechanism between the two specimens appear to be associated with a greater accumulation of material in the central zone of the specimen following collapse.

Although the distinction between X-type and Y-type fracture patterns is useful for describing the dominant failure paths, it must be emphasised that these classifications naturally arise from qualitative observation of the post-collapse morphology. The transition between a diagonal–diagonal propagation (X-type) and a diagonal–vertical propagation (Y-type) cannot be quantified in a strict geometric sense without introducing arbitrary metrics that would add little to the interpretation of the deformation mechanism. What is relevant from a mechanical standpoint is that the presence or absence of nodal spheres influences how and where the collapse initiates, thereby modifying the preferential trajectories along which the struts fail.

The incorporation of nodal spheres increases the local relative density in the central region of the unit cell, and this added mass alters the internal stress distribution during compression. By reducing stress concentration at the re-entrant angles and promoting a more gradual transfer of load toward the nodes, the structure can accommodate greater deformation before the onset of unstable fracture. Consequently, the directionality and sequence of failure become sensitive to the modified stress field, and the deformation mechanism transitions differently between bending-dominated and stretch-dominated responses. In this way, the nodal spheres play an active role in regulating the collapse process and shaping the resulting fracture pattern.

Subsequent fractures in both types of structures occur in the cells closest to the upper and lower plates. Following the fracture of these cells, the process of densification is initiated. Thus, the analysis of the mechanical behaviour of specimens shows a mixed deformation mechanism. At the initial stage, the structural response is governed by bending mechanisms, due to its topology. Once the maximum stress is reached (corresponding to the compressive strength of the structure) the behaviour transitions to a stretch-dominated regime.

**Figure 11:**
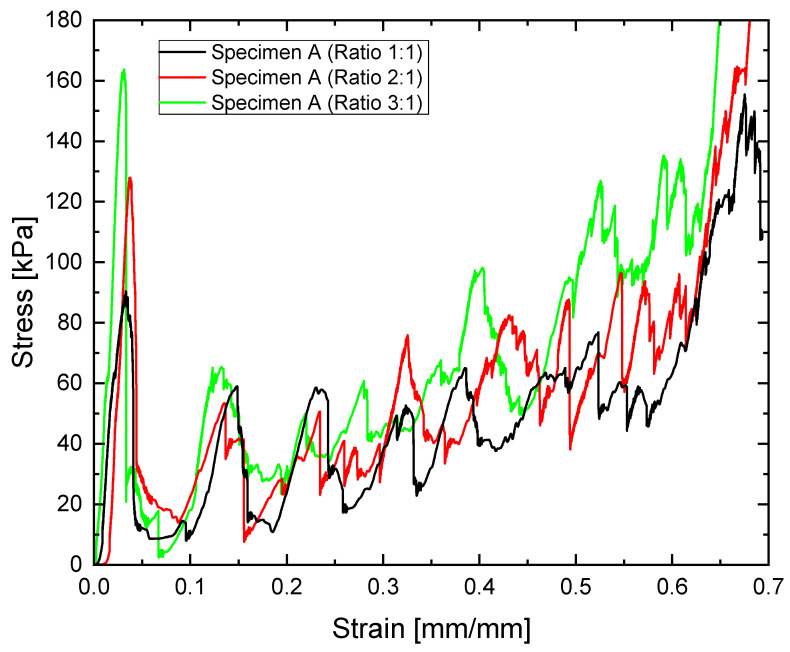
Comparison of different compression tests for specimen A corresponding to different node/strut ratios (1:1, 2:1, 3:1).

Figure 11 shows a comparison of the stress–strain curves in different metamaterials under in-plane compression. Experiments show that all metamaterials exhibit a primary stress peak, followed by a stress plateau. This plateau shows fluctuations in stress value, as reflected by several peaks in this region associated with stretch-dominated behaviour. Additionally, the curves show a sharp increase in stress, indicating the onset of densification. This behaviour and the presence of a similar number of peaks are observed in all analysed specimens, both with and without nodal spheres.

**Figure 12:**
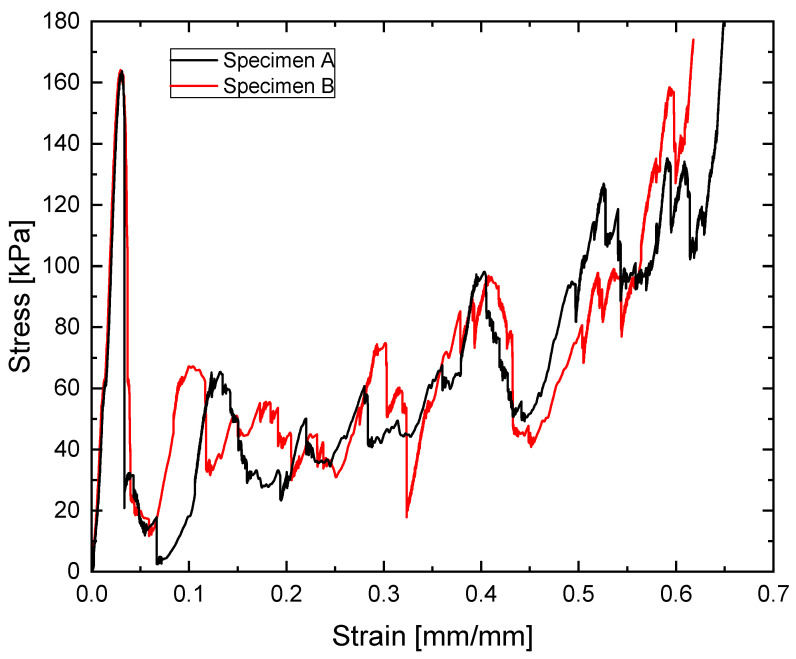
Stress–strain curve of compression tests of two specimens with nodal spheres of 3 times the diameter of the struts.

As an illustration of the reproducibility of the tests, the compression test results of two specimens corresponding to a 3:1 ratio, Specimen A and B, are presented in Figure 12.

Figure 11 shows that auxetic structures obtained with the dodecahedral cell model and two re-entrant angles at a ratio of 1:1 exhibit compressive behaviour with a maximum value of around 90 kPa. Densification of the material can be estimated to occur at a unit strain of 0.7 mm/mm.

For specimens with nodal spheres twice the diameter of the struts, a stress peak of around 130 kPa is observed. Densification strain is still observed at unit strain values of around 0.65 mm/mm.

Specimens with the largest nodal spheres demonstrate optimal RC values. Their maximum peak strain values are approximately 165 kPa. However, the densification strain remains at around 0.65 mm/mm, consistent with the other specimens tested.

**Figure 13:**
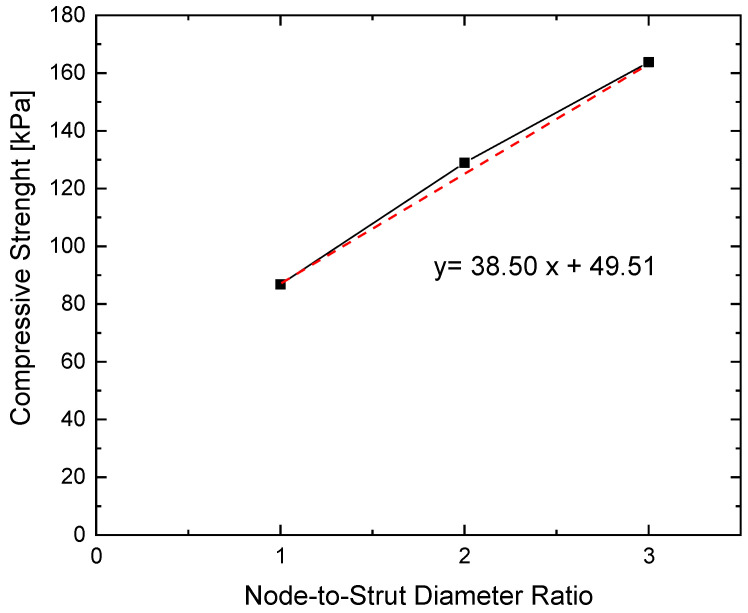
Relation between compressive strength and node-to-strut diameter ratio for analysed metamaterials. The red dashed line corresponds to the linear regression fit.

Figure 13 shows a graphical representation of the relationship between compressive strength and the ratio of nodal sphere to strut diameter for the specimens. The compression strength values show a linear relationship with the node/strut ratio.

So, a linear relationship can be established by approximating the equation of a straight line for the compressive strength, with a coefficient of determination of R^2^ = 0.997. The relationship obtained corresponds to Equation (2):

(2) $$R_{c}=38.50\times \left(\frac{\emptyset node}{\emptyset strut}\right)+49.51$$

In addition to determining the compressive strength, it is critical to determine the energy absorbed during the test for the application of these materials. Thus, the absorbed energy was determined as the area under the compression curve until the densification deformation value was reached. Regarding densification strain (ε_D_), there are several methods by which its value can be established, four of which stand out in particular [24]. One option is to use a fixed strain value of typically 40% or 50% [46,47]. Although this approach allows comparison between different studies, it does not consider the nature of the stress plateau at the specific strain value. An alternative approach involves the utilisation of an energy-efficient method, wherein the strain at the point of maximum energy efficiency is defined as ε_D_ [48,49,50]. This method has been found to be particularly effective when the plateau exhibits minimal hardening behaviour prior to a change in slope associated with densification. A third approach uses compressive strength as the threshold stress. If stress exceeds this threshold after the initial maximum, the definition of ε_D_ is implemented [51,52]. A fourth approach involves using the intersection of the slopes of the plateau and the densification region to define ε_D_ [43,53].

As the compressive strength has already been determined, the third method will be used to define densification deformation. This reduces the number of calculations required and takes the stress plateau into account. Thus, by drawing a horizontal line from the maximum stress peak, the stress threshold is established, and the densification strain can be determined.

**Figure 14:**
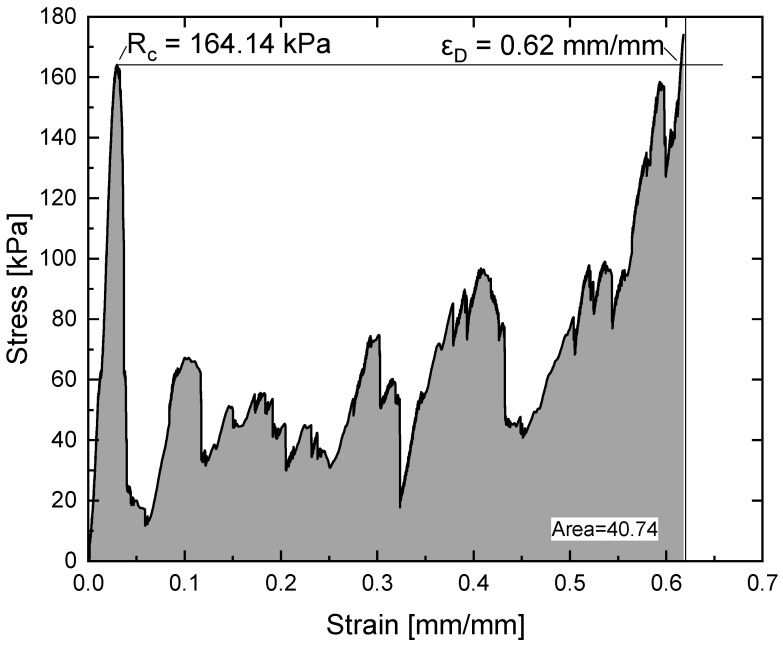
Absorbed energy calculated as area under stress–strain curve of specimen with ratio 3:1.

Figure 14 shows the calculation of the area under the compression curve obtained for the specimen with a 3:1 ratio. In all different types of metamaterials, the absorbed energy was calculated in the same way.

The elastic modulus of the metamaterial can be obtained by calculating the slope of the curve in the elastic zone, which is located before the first peak and covers approximately 1–2.5% strain.

The energy absorbed per unit mass can be quantified by calculating the area under the stress–strain curve before densification and then dividing this by the material’s density (i.e., PA12). To enable comparisons, specific energy absorption (SEA) is used, which is expressed in J/g and calculated according to Equation (3).

(3) $$SEA=\frac{\int_{0}^{{ɛ}_{D}}\sigma dɛ}{\rho },$$

To enable calculation of the specific energy absorption, the density of sintered PA12 was preliminarily estimated to be 894.90 kg/m^3^.

The results obtained from the compression tests on the remaining specimens are summarised in Table 1.

Analysing the results in Table 1, we can see that the compressive strength of structures containing nodal spheres twice the size is 48.53% higher than that of metamaterials without nodal spheres. For specimens containing nodal spheres three times the size, the increase in compressive strength is even greater at 88.70%. This suggests that introducing nodal spheres reduces stress concentrations.

Regarding the apparent Young’s modulus (E), specimens with spheres twice the diameter exhibit a 35.71% increase in stiffness, while those with spheres three times the diameter show a 45.63% increase.

Regarding specific energy absorption, specimens with a 2:1 sphere-to-strut ratio show a slight increase of 19.58%, whereas those with a 3:1 ratio show a significant increase of 59.04%. Therefore, the presence of nodal spheres enhances this property rather than deteriorating it.

To enable more accurate comparisons between different metamaterials, the obtained mechanical values and specific energy absorption have been normalised by the relative density of each structure, since mechanical properties are proportional to relative density. This eliminates the effects of mass and density, enabling the specific influence of the nodal spheres to be quantified.

First, the relative density of each metamaterial must be calculated using Equation (4):

(4) $$\rho_{R}=\frac{\rho *}{\rho }$$

where

ρ* is the macroscopic density, defined as the ratio between the mass of the structure and the volume of the prism enclosing the cellular material;

*ρ*′ is the density of the material used to manufacture the cell.

Consequently, the relative densities of the metamaterials with strut-to-node ratios of 1:1, 2:1, and 3:1 are 9.94%, 10.91%, and 15.37%, respectively. The previously calculated results, normalised by relative density, are presented in Table 2.

When the compressive strength results are normalised by the corresponding relative density, the value for specimens with a sphere-to-strut diameter ratio of 2:1 increases by 35.33%. For specimens with a ratio of 3:1, however, the increase is only 22.04%. In other words, when the effect of relative density is eliminated and the results are compared, it is evident that specimens with a 2:1 ratio achieve a higher compressive strength than those without nodal spheres or those with spheres three times the size. The latter show an increase of 10.89%.

Conversely, when the effect of mass is removed from the apparent Young’s modulus results, the increase for structures with nodal spheres with a diameter twice that of the struts decreases from 35.71% to 17.35%. For specimens with a 3:1 ratio, the Young’s modulus normalised by relative density decreases by 6.18% because using nodal spheres with diameters three times that of the struts significantly increases the relative density of the metamaterials.

Eliminating the influence of relative density results in an increase in specific energy absorption of 8.94% for the 2:1 specimens and 2.86% for the 3:1 specimen. These values are far lower than the un-normalised increases and are not statistically significant. However, they confirm that energy absorption is maintained or slightly improved rather than decreasing.

Overall, the 3:1 specimen demonstrate a substantial increase in mass, consequently leading to a higher relative density. This is because the nodal spheres are significantly larger, improving their mechanical properties compared to those of the other tested specimens. However, when the influence of relative density is removed to isolate that of the auxetic cell itself, it becomes clear that tripling the nodal sphere diameter does not improve the properties beyond those achieved with spheres measuring half the strut diameter. Conversely, excessive increases in sphere size reduce the structure’s effectiveness as a metamaterial due to the rise in mass and density, causing the mechanical properties to become more dependent on factors other than the unit cell’s intrinsic auxeticity.

**Figure 15:**
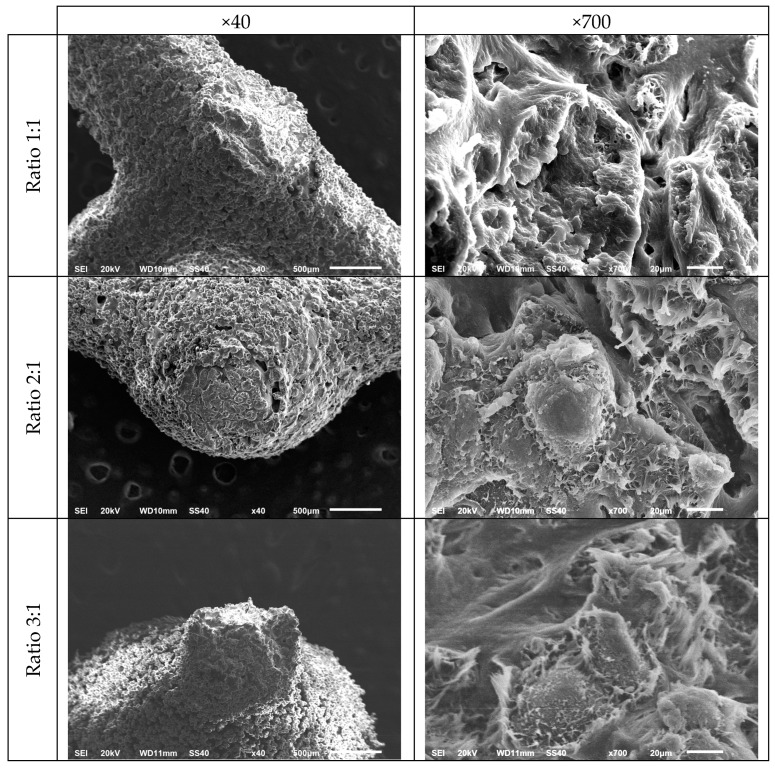
Scanning electron microscopy images of the compression fracture surfaces of three different specimens (Ratio 1:1, Ratio 2:1 and Ratio 3:1) at 40× and 700× magnification.

To analyse the compression fracture surfaces, a study using SEM was carried out. Figure 15 shows micrographs taken at 40× and 700× for the specimens corresponding to ratios of 1:1, 2:1 and 3:1. Prior to imaging, the fractured specimens were examined macroscopically and the key force-bearing regions (specifically the strut–node interfaces, where stress concentration is expected during compression) were intentionally selected for detailed SEM observation. In addition, a random sampling approach was applied, selecting multiple fracture locations from each specimen to avoid bias associated with a single local failure event.

Figure 15 shows that, during compression testing, fracture in the metamaterial specimens originates and propagates preferentially from the interfaces between the struts and nodal spheres. This behaviour suggests that these regions act as critical stress concentration zones, likely due to the degree of coalescence between sintered particles, facilitating crack initiation. As can be seen in the SEM micrographs, the fracture surface of all the specimens examined exhibits features that are typical of ductile deformation. Regions with the formation of fibrillar structures can also be observed, indicating a plastic deformation process prior to complete fracture. Therefore, the morphological examination reveals fracture morphology consistent with typical ductile fracture, corresponding to PA12.

## 4. Conclusions

This study investigated the influence of nodal spheres on the mechanical and energy absorption performance of PA12-based auxetic metamaterials fabricated via selective laser sintering. Scanning electron microscopy (SEM) observations revealed no significant differences in the fracture zones after compression testing across the different configurations examined. All specimens exhibited features characteristic of ductile fracture, with failure consistently initiating at the strut–node interface.

Compression testing showed that the mechanical behaviour of all specimens (with and without nodal spheres) exhibits a mixed deformation mechanism. Initially, the structural response is governed by bending mechanisms due to the structure’s topology. Once the maximum stress is reached, corresponding to the structure’s compressive strength, behaviour transitions to a stretch-dominated regime.

By systematically varying the node-to-strut diameter ratio (1:1, 2:1 and 3:1), the effects on stiffness, compressive strength and energy absorption under uniaxial compression were evaluated. The incorporation of nodal spheres markedly enhanced both stiffness and compressive strength, with improvements of up to 88.70% achieved for the 3:1 configuration. However, when normalised by relative density, the 2:1 configuration demonstrated the most efficient performance, yielding a 35.33% increase in specific compressive strength while maintaining superior energy absorption with only a modest increase in mass.

These findings confirm that introducing nodal spheres does not compromise the auxetic response; rather, it strengthens structural integrity and improves overall performance. The results provide valuable insights into the design of next-generation mechanical metamaterials, emphasising the importance of nodal reinforcement in optimising energy absorption and mechanical efficiency. The mechanical trends identified in this research demonstrate the potential of PA12-based auxetic metamaterials for engineering applications requiring lightweight structures with enhanced energy absorption. The demonstrated improvements in stiffness and specific strength position these architectures as promising candidates for use as impact energy absorbers in protective housings, crashworthy components and damping elements in lightweight mechanical systems. Furthermore, their favourable strength-to-weight ratio suggests applicability in weight-sensitive industries, such as in the manufacture of components for wind turbines and tidal energy generators, and in other renewable energy systems, where structural efficiency and resilience are critical.

However, the scope of this study is limited by its exclusive reliance on quasi-static uniaxial compression tests. The absence of dynamic loading or impact testing restricts our understanding of how nodal reinforcement influences rate-dependent behaviour, which is a key aspect for real-world energy absorption applications. Furthermore, fatigue performance was not evaluated, leaving unanswered questions regarding the long-term structural durability of these materials under cyclic loading.

Future research will therefore focus on experimentally assessing dynamic impacts, cyclic fatigue behaviour and strain-rate sensitivity in order to more accurately characterise the performance envelope of these metamaterials. Expanding this study to include comparisons with alternative polymers, as well as metallic auxetic structures, will further clarify the advantages and disadvantages of incorporating nodal spheres. Such investigations will support the development of robust design guidelines tailored to specific engineering environments and loading conditions.

## Figures and Tables

**Table 1:** Results of the compression tests of the metamaterials.

Specimen	R_C_ [kPa]	E [kPa]	ε_D_ [mm/mm]	Area [kPa]	SEA [J/Kg]
A (Ratio 1:1)	89.92	5058.10	0.63	26.83	29.98
B (Ratio 1:1)	83.69	4942.89	0.67	26.01	29.06
Mean	86.81	5000.50	0.65	26.42	29.52
Deviation	4.41	81.47	0.03	0.58	0.65
A (Ratio 2:1)	129.95	6936.70	0.66	30.71	36.28
B (Ratio 2:1)	127.93	6635.61	0.64	32.47	34.32
Mean	128.94	6786.16	0.65	31.59	35.30
Deviation	1.43	212.90	0.01	1.24	1.39
A (Ratio 3:1)	163.48	7336.97	0.65	42.02	46.95
B (Ratio 3:1)	164.14	7227.32	0.62	40.74	45.52
Mean	163.81	7282.15	0.64	41.38	46.95
Deviation	0.47	77.53	0.02	0.91	1.01

**Table 2:** Results of the compression tests of the metamaterials normalised by relative density.

Specimen	R_C_/*ρ_R_* [kPa]	E/*ρ_R_* [kPa]	SEA/*ρ_R_* [J/Kg]
A (Ratio 1:1)	904.63	50,886.32	301.62
B (Ratio 1:1)	841.95	49,727.26	292.40
Mean	873.29	50,306.79	297.01
Deviation	44.32	819.58	6.52
A (Ratio 2:1)	1191.11	63,581.12	332.57
B (Ratio 2:1)	1172.59	60,821.36	314.54
Mean	1181.85	62,201.24	323.56
Deviation	13.10	1951.45	12.75
A (Ratio 3:1)	1063.63	47,735.65	305.50
B (Ratio 3:1)	1067.92	47,022.25	296.19
Mean	1065.78	47,378.95	305.50
Deviation	3.03	504.45	6.58

## Data Availability

The original contributions presented in this study are included in the article. Further inquiries can be directed to the corresponding author.
